# Machine learning-based predictive model for immune checkpoint inhibitors response in gastrointestinal cancers

**DOI:** 10.3389/fmed.2025.1631011

**Published:** 2025-10-17

**Authors:** Yufan Lv, Qingbin Wang, Huiting Xu, Jing Dai, Yongchang Wei

**Affiliations:** ^1^Department of Radiation and Medical Oncology, Zhongnan Hospital of Wuhan University, Wuhan University, Wuhan, China; ^2^Hubei Key Laboratory of Tumor Biological Behaviors, Zhongnan Hospital of Wuhan University, Wuhan University, Wuhan, China; ^3^Department of Hematology, Zhongnan Hospital of Wuhan University, Wuhan University, Wuhan, China; ^4^Hubei Cancer Hospital, Wuhan, China

**Keywords:** predictive model, immune checkpoint inhibitors, treatment, gastrointestinal malignancies, machine learning

## Abstract

**Introduction:**

Gastrointestinal (GI) cancers present significant clinical challenges characterized by dismal survival outcomes and suboptimal prognoses. Currently, only partial indicators are available to predict the response of immunotherapy. A critical gap remains in the development of models capable of accurately predicting response rates to immunotherapy regimens. In this study, we developed a machine-learning (ML) model based on factorial, molecular, demographic, and clinical data to predict the response rate.

**Methods:**

This multicentre retrospective study analyzed the clinical data of 506 patients, comprising 352 cases collected from Zhongnan Hospital of Wuhan University and Hubei Cancer Hospital, along with 154 cases obtained from the publicly available dataset of Memorial Sloan-Kettering Hospital. We used 14 features as input features, such as the patient’s basic status, biochemical test results, and genetic test results. Eight ML methods were employed to build predictive models. Through rigorous validation using seven discriminative performance metrics (accuracy, precision, recall, F1-score, ROC-AUC, PR-AUC, and Brier score), the eXtreme Gradient Boosting (XGBoost) algorithm demonstrated superior predictive capability. Model interpretability was subsequently enhanced through Shapley Additive explanations (SHAP) analysis to elucidate feature contributions.

**Results:**

We selected XGBoost with the best predictive performance to predict response (AUC: 0.829 [95% CI: 0.72–0.91], accuracy: 78.43%, sensitivity: 86.67%, specificity: 72.31%). The Delong test and calibration curve indicated that XGBoost significantly outperformed the other models in prediction. The SHAP values indicate that chemotherapy contributes the most to the model’s predictive accuracy (contribution score = 0.28), while Ki-67 exhibits the lowest contribution rate (0.01). In addition, the study showed that chemotherapy, higher hemoglobin (HGB), body mass index (BMI), age, lower neutrophil-to-lymphocyte ratio (NLR), and tumor stage positively influenced the output of the model.

**Conclusion:**

Interpretable XGBoost models have shown accuracy, efficiency, and robustness in determining the association between input features and response rates. Among the input features, chemotherapy and tumor stage played the most important role in the prediction model. Due to the varying efficacy of ICIs in gastrointestinal cancers, personalized predictive models can greatly assist clinical decision-making. This model fills this gap in clinical practice and can provide more precise support for personalized treatment and risk avoidance.

## 1 Introduction

Gastrointestinal (GI) cancers are a group of diseases that seriously endanger the health of human beings, including esophageal cancers (EC) (1), gastric cancers (GC) (2)and colorectal cancers (CRC) (1, 3, 4). In recent years, the global morbidity and mortality of GI cancers have gradually increased and shown a trend of rejuvenation (5, 6). GI cancers are characterized by inconspicuous symptoms, high malignancy degree and propensity for metastasis. These pathophysiological characteristics collectively pose substantial challenges for clinical management and therapeutic intervention (7).

In recent years, immunotherapy has emerged as a transformative therapeutic paradigm, revolutionizing the treatment landscape for GI cancers (8, 9). Immune checkpoint inhibitors (ICIs) have achieved revolutionized success in hematological malignancies, yet their clinical application in GI cancers has yielded paradoxically limited therapeutic efficacy (10, 11). It has been well documented that the rate of clinical benefit in patients with GI cancers is low when ICIs are used alone (9, 12). Therefore, ICIs are usually combined with chemotherapy, radiotherapy, and targeted therapy in the treatment regimen of GI cancers (13, 14). Currently, some indicators such as tumor mutational burden (TMB) (15–17), microsatellite instability (MSI) (18–20), and PD-L1 expression (21) can initially assess the efficacy of ICIs. However, the response to therapy varies widely among patients with GI cancers. A model to predict response to combination therapy is presently lacking.

Machine learning (ML) is an important branch of artificial intelligence that has already achieved significant results in the medical field (22, 23). Currently, many studies have used ML methods to predict the prognosis of malignant tumors. However, there are still few studies on prediction models constructed by ML in GI cancers. In this study, we constructed a prediction model by ML to predict patients with GI cancers who are undergoing treatment based on ICIs. The model has a total of 14 input features, most of which have been shown to correlate with response rates. The variables incorporated included hemoglobin (HGB) (24), neutrophil-to-lymphocyte ratio (NLR) (25), sex (26), age (27), body mass index (BMI) (28, 29), cancer type, tumor stage (30), treatment modalities, and genetic test results (16). Taking whether to respond as the output target. In this study, a total of patients (*n* = 506) diagnosed with GI cancers were used as basic data. We found that most of the treatments received for GI malignancies (*n* = 352) in China were all combination therapies, so we chose the patients at Memorial Sloan-Kettering (*n* = 154) who were treated with immunotherapy alone as a control (4).

In this study, we developed a predictive framework to evaluate treatment response to ICI-based combination regimens in GI cancers. Firstly, we used eight ML methods (XGBoost, LightGBM, CatBoost, RandomForest, LR, KNN, Naive Bayes, and QDA) to comprehensively analyze the patients’ 14 input features before treatment. Subsequently, the model with the best predictive performance was selected and validated. Finally, the implementation of Shapley Additive exPlanations (SHAP) to quantify feature contributions and visualize non-linear relationships through summary plots and dependence analysis.

## 2 Methods

### 2.1 Patient data description

**Figure 1:**
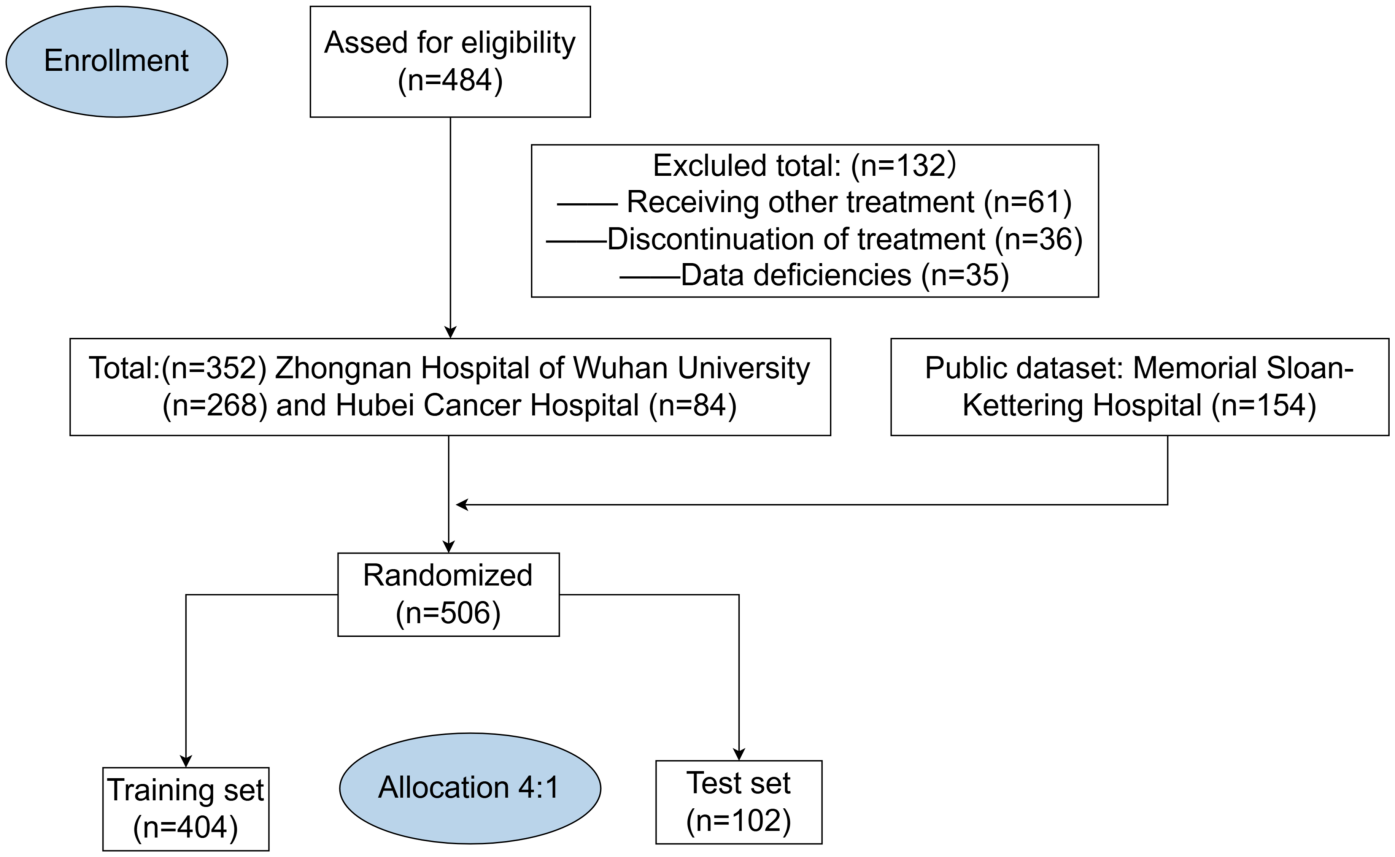
Patient screening and enrollment flowchart.

This multicentre retrospective study analyzed the clinical data of 506 patients, comprising 352 cases collected from Zhongnan Hospital of Wuhan University and Hubei Cancer Hospital, along with 154 cases obtained from the publicly available dataset of Memorial Sloan-Kettering Hospital (4). All MSK data are available online (https://www.ioexplorer.org). The inclusion criteria were as follows: (1) pathological diagnosis of gastrointestinal malignancy; (2) age ≥18 years; (3) having received at least four cycles of immunotherapy. The exclusion criteria were as follows: (1) having a primary or secondary history of cancer; (2) receiving traditional Chinese medicines, targeted therapies, or biologic therapies in the cycle of immunotherapy; (3) lack of follow-up information and clinical data. Patients initially selected for this study were those diagnosed with GI malignancies in 2021–2024 (*n* = 484), all of whom received at least four cycles of immunotherapy in the hospital. Subsequently, we retrospectively analyzed the clinical data of these patients. We excluded patients who had undergone targeted or biologic therapies during immunotherapy cycles (*n* = 61), and we further excluded patients who dropped out of treatment or died before completing four cycles of treatment (*n* = 36). At last, we excluded patients who were missing important basic clinical data (*n* = 35). After excluding all non-compliant patient data, we ultimately completed data collection from two Chinese hospitals (*n* = 352) (Figure 1).

### 2.2 Basic patient information and clinical data

We recorded basic health information by reviewing the nursing records before the first immunotherapy cycle, which included age, gender, and BMI. BMI was calculated as weight (KG) divided by the square of height (m^2^). All clinical blood test results were within 3 days before the first immunization cycle. NLR was calculated as absolute neutrophil count (per nanoliter) divided by absolute lymphocyte count (per nanoliter). Hemoglobin (HGB) was expressed in units of g L^- 131^. We documented tumor type, ICB drug class, and other treatments during the ICB treatment cycle by looking at physician-recorded cases. Drug class: the patients’ immunotherapy regimens were stratified into two cohorts: monotherapy with either PD-1/PD-L1 inhibitors or CTLA-4 inhibitors versus dual-agent immune checkpoint blockade combining both modalities. Cancers were staged according to the American Joint Committee on Cancer, 8th edition (31).

### 2.3 Genetic testing

Since numerous studies confirm that TMB is closely related to MSI (32, 33), we decided to choose MSI stability as an input feature (34). MSI: stable (0 ≤ MSI score < 3), uncertain (3 ≤ MSI score < 10), and unstable (10 ≤ MSI score). In the ML model, we used two groups for MSI status: MSI unstable versus MSI stable/indeterminate. For patients with MMR deficiency, we further conduct genetic sequencing to confirm the MSI status. Gene mutations: it is well documented that HER-2 and K-RAS genes play an important role in GC and CRC and determine the prognosis of patients (35). Therefore, we incorporated the mutation status of these two genes as one of the input features in our predictive model. The mutation status of MSI, KRAS, and HER2 genes was determined using next-generation sequencing (NGS). To reduce patient costs and improve the accuracy of genetic testing, targeted sequencing panel approaches were employed for all analyses.

### 2.4 Ki-67 and CPS

Both CPS and Ki-67 scores were assessed through immunohistochemistry (IHC). Pathologists determined the scores by observing the percentage of Ki-67 and PD-L1 positive cells. In our study, the Ki-67 input score was based on the percentage of Ki-67 positive cells as documented in the pathology report. For PD-L1 expression (CPS score), a score greater than or equal to 1 was considered positive.

IHC Staining: tissue sections were dewaxed by immersing in xylene twice for 10 min each, followed by hydration in an alcohol gradient. Antigen retrieval was performed by placing the tissues in citrate sodium repair solution. The sections were incubated with the desired antibodies overnight at 4 °C. The next day, rapid color development was achieved using DAB, and expression levels were estimated using IHC scoring. Specific antibody catalog numbers and dilution ratios are provided in Supplementary Table 3.

### 2.5 Response

We reviewed the doctor’s case records to determine the patient’s treatment outcome. Response was based on Response Evaluation Criteria in Solid Tumors (RECIST) v1.1 (36). The primary outcome of the study was an assessment of overall treatment efficacy. Complete response (CR), partial response (PR), and stable disease (SD) were categorized as treatment effective, and progressive disease (PD) was categorized as treatment ineffective.

### 2.6 Model training

Data division: we divided the data of 506 patients into training (80%) and test (20%) sets using stratified random sampling, ensuring that both response rate and hospital distribution were balanced between the training set and test set.

Parameter selection: Hyper-parameter optimization was performed using the Optuna framework (37). For each model, we defined a search space (For XGBoost: We set the range of the n_estimators’ parameter from 20 to 200, the max_depth parameter from 3 to 12, and the learning_rate parameter from 0.001 to 0.3.). The optimization objective was to maximize the mean cross-validated AUC under a five-fold stratified cross-validation scheme on the training set. Each Optuna trial was allowed to run for up to 200 iterations, and the trial with the best validation AUC was chosen. The final model was retrained on the entire training set using the best parameters. All eight ML models were trained following this procedure. Random seeds were fixed to ensure reproducibility (random seeds for python and numpy were set to 42).

### 2.7 ML methods and SHAP analysis

A total of eight ML methods were used in this study which are XGBoost, LightGBM, CatBoost, RandomForest, LR, KNN, Naivebayes, and QDA. We used hyperparameter optimization to optimize the performance of each ML model (38). Important metrics we used to evaluate the performance and generalization of ML models include area under the ROC curve (AUC), PR-AUC, accuracy, sensitivity, Specificity, and so on (39). From these, the best-performing model was selected and validated for analysis. SHAP is one of the most commonly used interpretability tools (40). In this study, we visualized the analysis by using the SHAP method to work out the contribution of each feature to the model output.

### 2.8 Handling of missing values

For the treatment of missing values, more than 35 % of the missing features were not included in our study. For models such as XGBoost, LightGBM, and CatBoost, the built-in mechanisms for handling missing data values of these models eliminate the need for manual preprocessing. In contrast, for models including LR, KNN, Naivebayes, QDA, and Random Forest, we employed the Multiple Imputation by Chained Equations (MICE) method to impute missing values.

### 2.9 Statistical analysis

All analyses were performed using IBM SPSS software (version 26.0), R software (version 4.0.5), and the Python scikit-learn package (version 1.6.0). Response rates were compared by chi-square test and Fisher’s exact test, we use the De-long test to compare the AUC of the different models. *p* < 0.05 was statistically significant. For full implementation details of this study, please refer to the source code repository: https://github.com/wangqingbin/ML-Digestive-Cancer.

## 3 Results

### 3.1 Baseline characteristics of the patient

**Figure 2:**
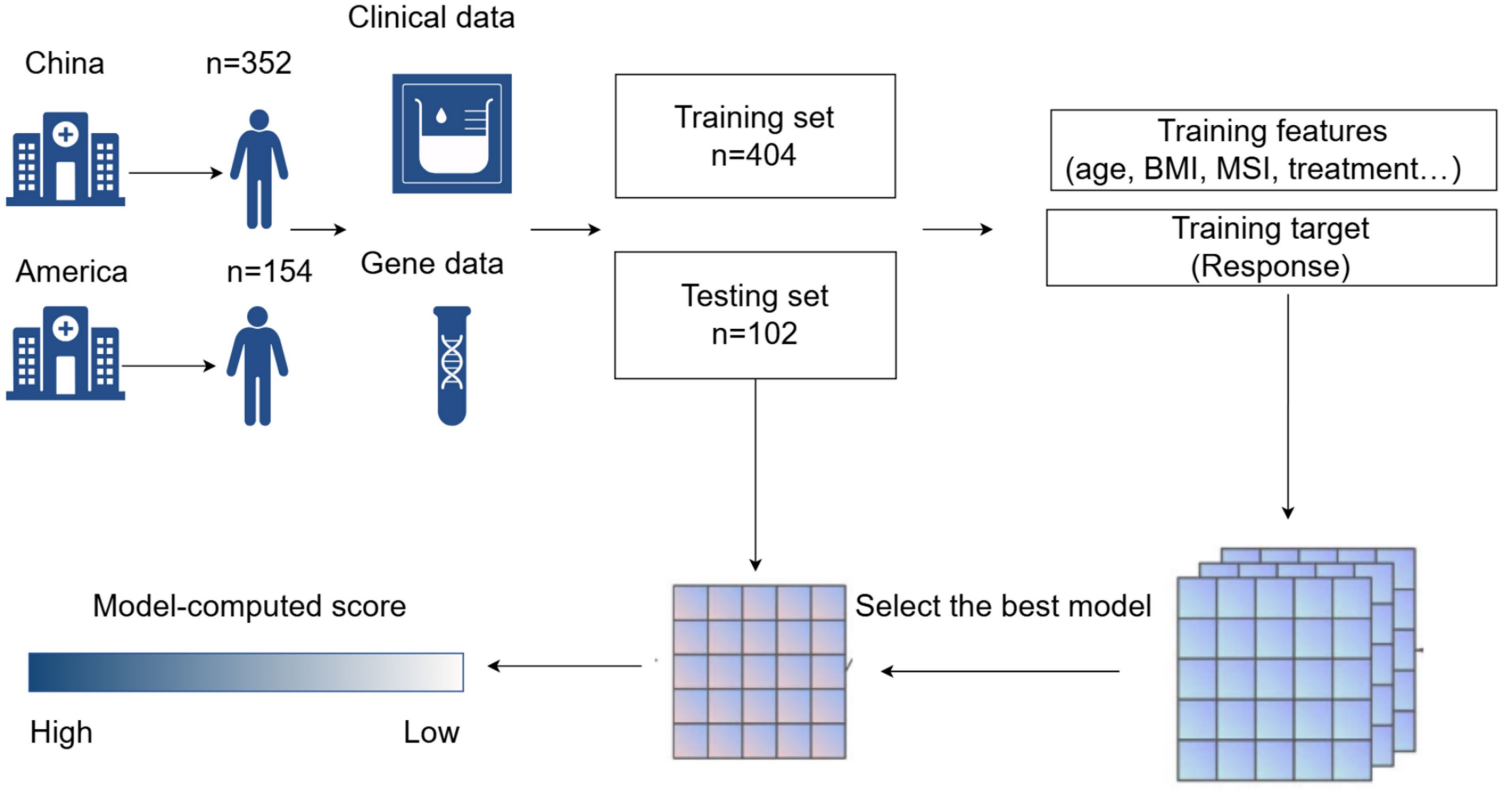
Process diagram for model construction.

Figure 2 illustrates the process of participant selection and study design. The basic characteristics of the 506 patients included in this study are shown in Table 1. The cohort was comprised of mostly males (65%), with a median age at diagnosis of 60 (IQR, 52–67) years. Of these patients, 44.5% had a history of surgery (patients with postoperative recurrence), median BMI was 22.65 (19.86–25.32). There were 127 (25.09%) patients diagnosed with EC, 228 (45.05%) with GC, and 151 (29.86%) with CRC. The total number of treatment responders was 300 (59.3%).

**Table 1:** Characteristics of patients in the study.

Characteristic	Total patients (*n* = 506)	Training set (*n* = 404)	Test set (*n* = 102)
Sex, *n* (%)			
Female	172	133	39
Male	334	271	63
Age, median, (IQR)	60 (52–67)	60 (52–67)	60 (53–68)
Cancer type, *n* (%)			
Esophageal	127 (25.09)	101 (25)	26 (25.49)
Gastric	228 (45.05)	182 (45.05)	46 (45.10)
Colorectal	151 (29.86)	121 (29.95)	30 (29.41)
Stage *n* (%)			
I-III	123 (24.4)	96 (23.76)	27 (26.47)
IV	383 (75.6)	308 (76.24)	75 (73.53)
Surgery history *n* (%)			
Yes	225 (44.5)	174 (43.07)	51 (50)
No	381 (55.5)	230 (56.93)	51 (50)
Response *n* (%)			
Yes	300 (59.3)	240 (59.41)	60 (58.82)
No	206 (40.7)	164 (40.59)	42 (41.18)

### 3.2 Machine prediction model

**Figure 3:**
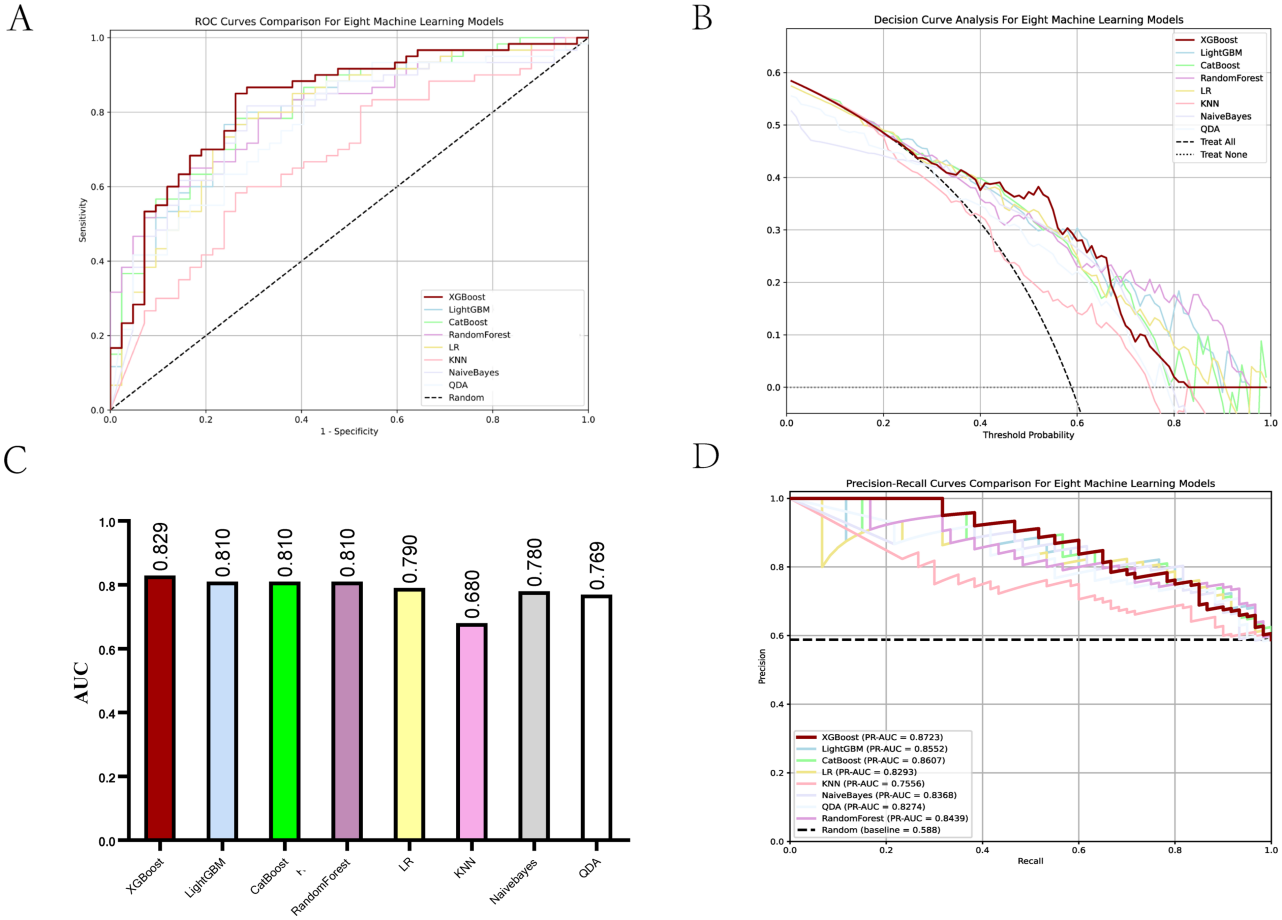
Evaluation of ML models. **(A)** ROC curves for all ML models. **(B)** Decision curves for all ML models. **(C)** AUC values for all ML models. **(D)** PR-AUC for all ML models.

**Figure 4:**
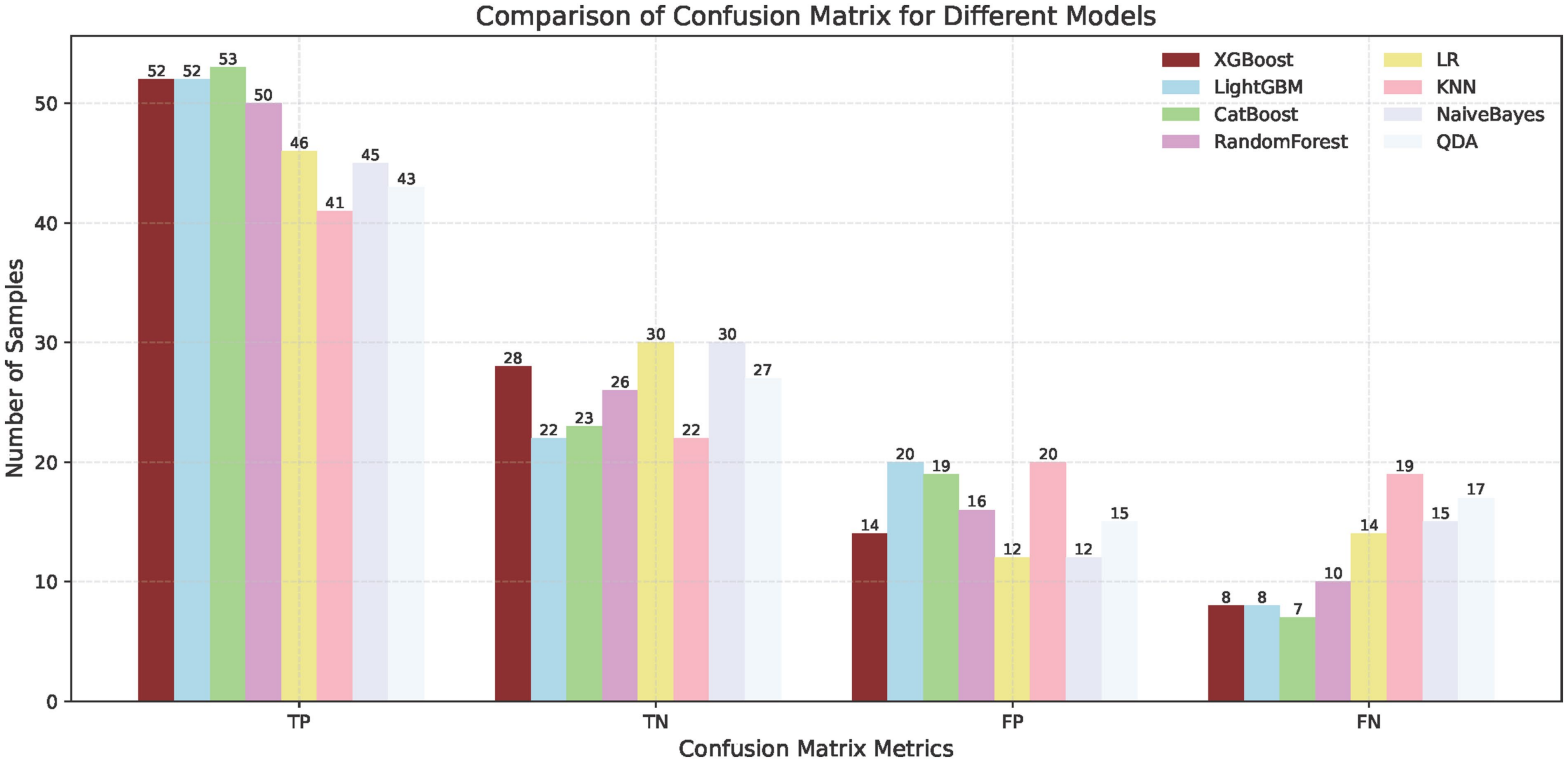
Confusion matrix for different models.

To predict the treatment response rate of patients with GI malignant tumors, we developed and trained eight ML models. The AUC curves of all of these models are shown in Figure 3A and the values of AUC are shown in Figure 3C. The decision curves of all models are shown in Figure 3B. The AUC value of XGBoost was 0.829 (95% CI: 0.73–0.91). The De-long test results suggested that the difference in the AUC between XGBoost and other ML models was statistically significant (*p* < 0.05). Given the imbalanced nature of our dataset, we incorporated the Precision-Recall AUC (PR-AUC) metric to comprehensively evaluate model performance beyond conventional ROC analysis, the Xgboost PR-AUC = 0.8723 (Figure 3D). Subsequently, we used metrics such as accuracy, sensitivity, and specificity to evaluate the accuracy of all models (Table 2). We showed the number of true positives, true negatives, false positives, and false negatives predicted by each model further demonstrated in the form of Figure 4. XGBoost model achieved the best performance among these methods.

**Table 2:** Detailed parameters of each machine learning mode.

Model	Accuracy	Sensitivity	Specificity	PPV (%)	NPV (%)
XGBoost	78.43 (69.19–85.96)	86.67 (75.41–94.06)	66.67 (50.45–80.43)	78.79 (66.98–87.89)	77.78 (60.85–89.88)
LightGBM	72.55 (62.82–80.92)	86.67 (75.41–94.06)	52.38 (36.42–68.00)	72.22 (60.41–82.14)	73.33 (54.11–87.72)
CatBoost	74.51 (64.92–82.62)	88.33 (77.43–95.18)	54.76 (38.67–70.15)	73.61 (61.90–83.30)	76.67 (57.72–90.07)
RF	74.51 (64.92–82.62)	83.33 (71.48–91.71)	61.9 (45.64–76.43)	75.76 (63.64–85.46)	72.22 (54.81–85.80)
LR	74.51 (64.92–82.62)	76.67 (63.96–86.62)	71.43 (55.42–84.28)	79.31 (66.65–88.83)	68.18 (52.42–81.39)
KNN	61.76 (51.61–71.21)	68.33 (55.04–79.74)	52.38 (36.42–68.00)	67.21 (54.00–78.69)	53.66 (37.42–69.34)
Naivebayes	73.53 (63.87–81.78)	75 (62.14–85.28)	71.43 (55.42–84.28)	78.95 (66.11–88.62)	66.67 (51.05–80.00)
QDA	68.63 (58.69–77.45)	71.67 (58.56–82.55)	64.29 (48.03–78.45)	74.14 (60.96–84.74)	61.36 (45.50–75.64)

### 3.3 SHAP analysis and importance of features

**Figure 5:**
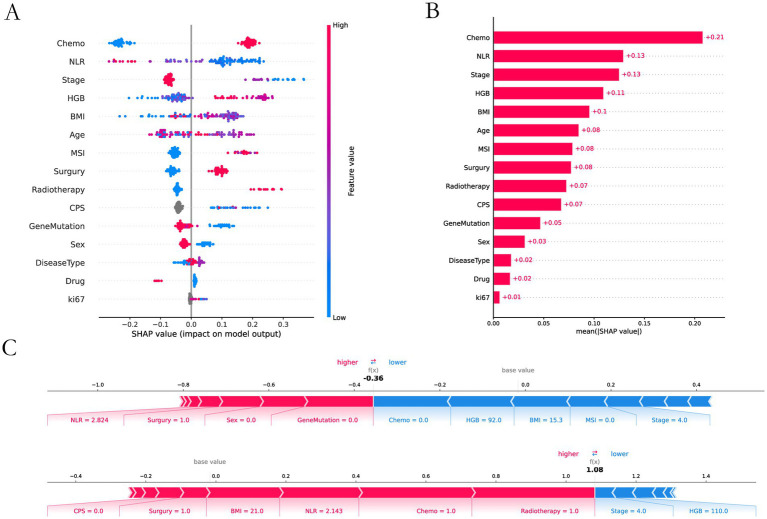
SHAP interpretability analysis. **(A)** Interpretable and analyzable swarm maps. **(B)** Contribution of each input feature. **(C)** Local interpretation of each input feature.

The feature importance analysis we performed on XGBoost by using an interpretable SHAP analysis approach (Figure 5). Chemotherapy scored highest in feature contribution, indicating the highest contribution to model accuracy. The lowest score was Ki67, indicating the lowest contribution to model accuracy.

### 3.4 Analysis of key risk factors

In the XGBoost-based feature importance analysis and SHAP analysis, treatment modality and tumor stage emerged as the two most influential features. We performed a detailed analysis of the relationship between these features and response rate. We analyzed the effects of different treatment modalities and different tumor stages on response rates (Table 3).

**Table 3:** Detailed analysis of important features.

Characteristic	Response, *n* (%)	No response, *n* (%)	χ ^2^	*p*
Treatment				
ICIs	118 (53.8)	102 (46.2)	16.96	<0.01
Chemo+ICIs	159 (72.6)	60 (27.4)		
Stage				
I–III	103 (83.7)	20 (16.7)	41.44	<0.01
IV	195 (50.90)	188 (49.10)		

## 4 Discussion

In recent years, ICIs have been widely used in the treatment of GI cancers (41). However, with the rise of immunotherapy, challenges have emerged. For example, the response rate remains relatively low and varies significantly among individuals in this field. How to enhance immune response rates and refine personalized immunotherapy strategies stands as a critical challenge in the field today. Therefore, we developed and trained eight ML models—XGBoost, LightGBM, CatBoost, RandomForest, LR, KNN, Naïve Bayes, and QDA—to analyze data from patients with GI cancers. Within our predictive framework, both the XGBoost and CatBoost classifiers demonstrated high predictive efficacy, achieving AUC values of 0.829 and 0.812, respectively. Further analysis revealed that the XGBoost classifier outperformed CatBoost in both accuracy and specificity metrics. Consequently, XGBoost proves to be a robust tool for accurately predicting the response of ICIs therapy. In short, these data indicate that our ML method can predict immunotherapy response rates in GI cancers with high accuracy prior to treatment.

From the baseline chart of patients, it can be seen that the incidence rate of GI cancers is much higher in men than in women, with the incidence rate reaching 65%, which may have a great relationship with factors such as smoking and drinking (42). In addition, the proportion of patients entering stage IV reaches 75.6%, which indicates that GI cancers are characterized by late detection. Most of the patients had already metastasized by the time they sought medical treatment.

We used 8 ML methods to construct the prediction model. XGboost, with an AUC value of 0.829 and a sensitivity of 0.8667, had the best prediction performance among these models. The SHAP explanation indicates that chemotherapy is the most significant predictive feature (contribution score = 0.28), which aligns with the clinical practice of chemotherapy serving as the cornerstone of GI cancer treatment. Mechanistically, this process likely involves multiple factors. Firstly, chemotherapy enhances tumor antigen presentation and T-cell-mediated cytotoxicity, thereby potentiating immunotherapy through “sensitization” effects (43). Secondly, combination therapies significantly mitigate the risk of tumor cells developing resistance to single-treatment modalities, thereby enhancing therapeutic efficacy through synergistic effects (44). The study by Ningchen et al. investigated the association between nutritional status and the efficacy of immune checkpoint inhibitor therapy in esophageal cancer. The research demonstrated that patients’ pretreatment HGB levels and BMI were significantly correlated with treatment effectiveness, and both served as independent prognostic indicators for survival outcomes (45). In our study, we found that a higher level of HGB and BMI significantly improved the therapeutic effect. In our predictive model, the feature importance of BMI and HGB was 0.14 and 0.15, respectively. Therefore, the patient’s baseline nutritional status positively influences the response rate to immunotherapy. In other studies, NLR is an important indicator of the degree of inflammation (25), and this was indirectly confirmed in our study. The higher the NLR ratio, the worse the outcome for the patients, which is probably related to the degree of inflammation in the patient’s body. In tumor staging, once a patient enters stage IV and metastasis occurs, the response rate will be greatly reduced. Once tumor metastasis occurs, the therapeutic efficacy of immunotherapy is significantly diminished. The MSI and PD-L1 expression are very important features to measure the efficacy of immunotherapy (21, 46), but our prediction model is a combination therapy model based on immunotherapy, and the MSI and PD-L1 expression does not have absolute importance in terms of the model’s contribution, and we speculate that in the combination therapy model. We speculate that in the combination therapy model, immunotherapy contribution is inherently low and assumes an adjunctive therapeutic role. Interestingly, age also plays an important role in the contribution of characteristics, and we found that the older the age, the higher the response rate, which we think may be related to the fact that young people have a fast basal metabolism, and tumors are more likely to progress and metastasize. In addition, gene mutations also contribute to treatment response rates, HER-2 positivity in GC and K-RAS mutations in CRC reduce response rates. Ki-67 is expressed in the nucleus. Once cells enter the quiescent G0 phase, Ki-67 undergoes rapid degradation, making its index value a reliable indicator of cellular proliferative activity (47). Paradoxically, while elevated Ki-67 levels correlate with accelerated tumor cell proliferation rates, this proliferation marker simultaneously demonstrates a strong positive association with chemosensitivity - tumors exhibiting high Ki-67 expression demonstrate enhanced responsiveness to chemotherapy and achieve superior treatment outcomes. This dual biological significance (pro-proliferative yet pro-chemosensitive) likely accounts for its low feature contribution rate (0.01) in our immunotherapy predictive model. In SHAP interpretability analysis, the treatment method and tumor stage are the two features with the highest contribution rates. Subsequently, we performed a deeper analysis of these two features. Table 3 shows that immunotherapy alone has a low response rate while combining immunotherapy with chemotherapy increases the response rate to 72.6%. Once the tumor reaches stage IV, the response rate drops dramatically, from 80.7 to 50.6%.

In recent studies, ML has shown significant potential in predicting the efficacy of immunotherapy. Hui Liu et al. developed a multimodal prediction model for immunotherapy of esophageal cancer, the study developed a predictive model for immunotherapy response in esophageal cancer by integrating pathology images, CT scans, and clinical data, achieving an AUC of 0.809 (48). Hong Wei Li et al. developed a predictive model for the efficacy of immunotherapy in gastric cancer, the study leveraged clinical data from 273 gastric cancer patients to construct predictive models for overall survival (OS) and progression-free survival (PFS) in response to immunotherapy, with a specific focus on patients’ nutritional status. The XGBoost model achieved an AUC of 0.723 in predicting treatment outcomes (49). Current studies have focused primarily on single cancer types rather than pan-GI malignancie. Our study addresses this gap by developing an interpretable ML framework to predict immunotherapy treatment responses across three major GI cancers: EC, GC, and CRC. Currently, clinical approaches for predicting immunotherapy responses still primarily rely on MSI status, TMB, or physicians’ subjective clinical expertise. However, the tumor immune microenvironment is extremely complex, and relying solely on any single detection method cannot accurately predict immunotherapy response rates. Therefore, it is imperative to develop personalized immunotherapy strategies for patients and build predictive models for immunotherapy efficacy. Therefore, our study constructs a predictive model incorporating multiple dimensions—including common nutritional status indicators, blood biochemical markers, imaging findings, and genetic testing results. All metrics utilized are readily obtainable in routine clinical practice, enabling more effective tailoring of personalized treatment plans for individual patients.

Our study holds significant implications for clinical practice in cancer therapy. First, chemotherapy remains the cornerstone of comprehensive cancer treatment, and combination regimens can substantially enhance response rates to immunotherapy. Second, for gastrointestinal malignancies, once patients progress to stage IV, the efficacy of immunotherapy declines markedly. Hence, early screening, detection, and intervention are critically important in clinical management. Additionally, patients’ systemic health status profoundly impacts immunotherapy outcomes—maintaining optimal nutritional status and controlling inflammatory responses are essential. Finally, traditional predictive biomarkers from genetic testing remain indispensable; notably, MSI status retains its irreplaceable role in forecasting immunotherapy responsiveness. In summary, the determinants of immunotherapy efficacy are multifaceted. To optimize therapeutic success, clinicians should adopt a holistic approach that integrates all relevant factors.

However, our study still has several limitations. While basic clinical characteristics including TNM staging, BMI, NLR, and HGB were assessed in 100% of patients, genetic testing was not performed in all cases. Specifically, out of a total of 506 patients with GI cancers, 381 underwent MSI testing; among 228 GC patients, 164 had HER-2 status evaluated; and among 151 CRC patients, 105 completed K-RAS testing. These missing data may have introduced bias that could potentially affect the accuracy of our predictive model. Furthermore, the lack of experimental validation remains a constraint, and additional experimental studies will be required to enhance the clinical applicability of our findings in future research. Furthermore, in our research, we split all the data into training and validation sets, but still lack an independent external validation set. To verify the accuracy of the model, we will need to use an additional independent external validation set for validation in the future.

## 5 Conclusion

XGBoost performed optimally with other ML methods in terms of modeling to predict response effects with clinical accuracy. Through comprehensive feature importance analysis, chemotherapy regimen and tumor staging parameters emerged as the most influential predictors, collectively accounting for 43% of the model’s predictive capacity (Shapley value analysis). We will further conduct continuous tracking analysis and interpretation of the selected features to validate and apply the prediction model for the treatment effectiveness of patients with GI cancers.

## Data Availability

The original contributions presented in the study are included in the article/Supplementary material, further inquiries can be directed to the corresponding authors.
