# Supplementary material for: Machine learning-based predictive model for immune checkpoint inhibitors response in gastrointestinal cancers
Source: Front Med (Lausanne). 2025 Oct 17;12:1631011. doi: 10.3389/fmed.2025.1631011 (PMC12575239; doi:10.3389/fmed.2025.1631011)
Supplement: Supplementary file 1 [file Table_1.DOCX]

**Supplementary table1 Hyperparameter Settings for Machine Learning Models**

| Model | Key Hyperparameters | Value Range / Settings | Notes |
| --- | --- | --- | --- |
| XGBoost | learning_rate, n_estimators, max_depth, subsample,  colsample_bytree, reg_alpha, reg_lambda | [0.01, 0.05, 0.1, 0.2]  [100, 200, 300]  [3, 5, 7, 9]  [0.6, 0.8, 1.0]  [0.6, 0.8, 1.0]  [0, 0.1, 1] | Optuna with 5-fold CV |
| LightGBM | learning_rate, n_estimators, max_depth, num_leaves, min_data_in_leaf, subsample | [0.01, 0.05, 0.1, 0.2]  [100, 200, 300]  [5, 10]  [31, 64, 128]  [20, 50, 100]  [0.6, 0.8, 1.0] | Optuna with 5-fold CV |
| CatBoost | learning_rate, iterations,  depth,  l2_leaf_reg | [0.01, 0.05, 0.1, 0.2]  [100, 200, 300]  [4, 6, 8, 10]  [1, 3, 5, 7] | Optuna with 5-fold CV |
| Random Forest | n_estimators, max_depth, min_samples_split, max_features | [100, 200, 300]  [10–30]  [2, 5, 10]  [auto, sqrt, log2] | Optuna with 5-fold CV |
| Logistic Regression | penalty,  solver | [‘l1’, ‘l2’]  ['liblinear', 'saga'] | Optuna with 5-fold CV |
| KNN | n_neighbors,  weights,  metric | [3–11]  [uniform, distance]  [euclidean, manhattan] | Optuna with 5-fold CV |
| Naive Bayes | alpha | [0.1, 0.5, 1.0] | Optuna with 5-fold CV |
| QDA | reg_param | [0.0, 0.1, 0.5, 1.0] | Optuna with 5-fold CV |
