# Supplementary material for: Machine learning-based predictive model for immune checkpoint inhibitors response in gastrointestinal cancers
Source: Front Med (Lausanne). 2025 Oct 17;12:1631011. doi: 10.3389/fmed.2025.1631011 (PMC12575239; doi:10.3389/fmed.2025.1631011)
Supplement: Supplementary file 2 [file Table_2.DOCX]

**Supplementary table2 Datasets comparison**

| **Details** | **Dataset1** | **Dataset2** | **Dataset3** |
| --- | --- | --- | --- |
| **Type** | Local dataset | Local dataset | Public dataset |
| **Access** | Upon request | Upon request | Freely available |
| **Hospital** | Zhongnan Hospital of Wuhan University | Hubei cancer Hospital | Memorial Sloan-Kettering Hospital |
| **Storage type** | Record | Record | Record |
| **Time** | 2021 through 2024 | 2023 through 2024 | 2015 through 2018 |
| **Number of samples** | 268 | 84 | 154 |
| **Type of features** | 14 | 14 | 16 |
| **Missing data** | Yes | Yes | No |
| **Distribution type** | Unbanlanced | Unbanlanced | Unbanlanced |
