# Supplementary material for: Machine learning-based predictive model for immune checkpoint inhibitors response in gastrointestinal cancers
Source: Front Med (Lausanne). 2025 Oct 17;12:1631011. doi: 10.3389/fmed.2025.1631011 (PMC12575239; doi:10.3389/fmed.2025.1631011)
Supplement: Supplementary file 3 [file Table_3.DOCX]

**Antibody Catalog**

| Target | Product information | Diluted concentration |
| --- | --- | --- |
| MMR | Abcam |  |
| MLH1 | Abcam #ab92312 | 1:1000 |
| MSH2, | Abcam #ab227941 | 1:1000 |
| MSH6 | Abcam #ab92471 | 1:1000 |
| PMS2 | Abcam #ab110638 | 1:1000 |
| PD-L1 | Abcam; #ab213564 | 1:300 |
| Ki-67 | Abcam; #ab15580 | 1:500 |
